# Seismic tomography of the area of the 2010 Beni-Ilmane earthquake sequence, north-central Algeria

**DOI:** 10.1186/2193-1801-3-650

**Published:** 2014-11-04

**Authors:** Issam Abacha, Ivan Koulakov, Fethi Semmane, Abd Karim Yelles-Chaouche

**Affiliations:** Centre de Recherche en Astronomie, Astrophysique et Geophysique, BP. 63, Bouzareah, Alger, Algeria; Trofimuk Institute of Petroleum Geology and Geophysics, SB RAS, Prospekt Koptyuga, 3, Novosibirsk, 630090 Russia; Novosibirsk State University, Novosibirsk, Russia

**Keywords:** Beni-Ilmane earthquake, Tellian chain, Aftershocks, Seismic tomography, Fluids

## Abstract

The region of Beni-Ilmane (District of M’sila, north-central Algeria) was the site of an earthquake sequence that started on 14 May 2010. This sequence, which lasted several months, was triggered by conjugate E–W reverse and N–S dextral faulting. To image the crustal structure of these active faults, we used a set of 1406 well located aftershocks events and applied the local tomography software (LOTOS) algorithm, which includes absolute source location, optimization of the initial 1D velocity model, and iterative tomographic inversion for 3D seismic P- and S-wave velocities (and the Vp/Vs ratio), and source parameters. The patterns of P-wave low-velocity anomalies correspond to the alignments of faults determined from geological evidence, and the P-wave high-velocity anomalies may represent rigid blocks of the upper crust that are not deformed by regional stresses. The S-wave low-velocity anomalies coincide with the aftershock area, where relatively high values of Vp/Vs ratio (1.78) are observed compared with values in the surrounding areas (1.62–1.66). These high values may indicate high fluid contents in the aftershock area. These fluids could have been released from deeper levels by fault movements during earthquakes and migrated rapidly upwards. This hypothesis is supported by vertical sections across the study area show that the major Vp/Vs anomalies are located above the seismicity clusters.

## Introduction

**Figure 1:**
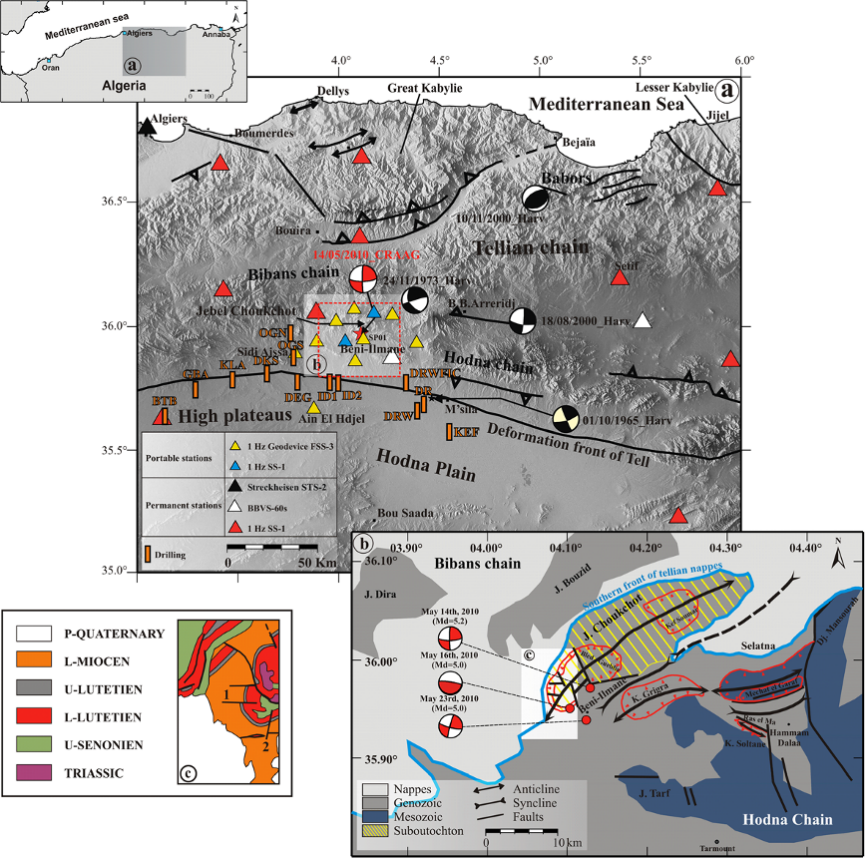
**Seismo-tectonic context of the 2010 Beni-Ilmane seismic sequence (from** **Yelles et al.** 2013 **)** **with the seismic networks.** The brown rectangles represent the drillings in the region and some among them attained around 4000 m of depth. The red dashed box shows the tomography area shown in Figures 3, 4 and 6.

Northern Algeria has been the location of many destructive earthquakes, including the recent 21 May 2003 strong (M_w_6.9) event at Boumerdes (e.g., Yelles et al. 2004). Several recent moderate seismic events have also occurred in this region, such as the 2006 Lâalam earthquake (M_w_5.2) (Beldjoudi et al. 2009) in the Kherrata fault system north of Setif and the 2006 Tadjena earthquake (M_w_5.0) (Beldjoudi et al. 2011). In addition, in 2010, an earthquake sequence occurred in the region of Beni-Ilmane (Yelles et al. 2013), a small village located in the southern part of the Tellian Atlas and about 200 km southeast of Algiers (Figure 1a). This earthquake sequence caused 3 fatalities and injured 170 people, and generated substantial damage to houses and local infrastructure in the epicentral area.

An earthquake sequence such as the 2010 Beni-Ilmane sequence is uncommon in Algeria in several respects. First, the sequence consisted of three main shocks of very similar magnitudes (5.0 < M_d_ <5.2) over a period of nine days in a localized area. Second, the sequence was characterized by strong aftershock activity (more than 24,000 events were recorded by a temporary seismic network during the first week of the sequence) that lasted for several months. Third, the earthquake sequence occurred in a region of lower seismic activity in northern Algeria, and more precisely between two geological transition zones; namely, the Bibans-Hodna Mountains and the Tellian Chain High Plateaus (Figure 1a).

Prior to the Beni-Ilmane sequence, the study region was characterized by low seismic activity, although some historical events have occurred here. The most notable event occurred close to the epicenter of the 2010 Beni-Ilmane event on 21 February 1960, and was of magnitude M5.6. This event generated similar macroseismic effects to those of the Beni-Ilmane event, and its maximum intensity was VIII (MSK) (Benouar 1994).

To understand the crustal structure in the Beni-Ilman region, and to enhance the accuracy of source locations there, we performed a tomographic inversion that uses the simultaneous determination of P- and S-wave velocity distributions as well as the relocation of aftershocks. Local earthquake tomography (LET) is a useful tool for imaging lateral heterogeneities in the upper crust based on the relationship between the pattern of P- and S-wave velocity anomalies and the clustering of aftershocks along active fault zones. LET has been used for studying the distribution of seismic velocity in several major fault systems, including the San Andreas Fault in the western US (e.g., Chiarabba and Amato 1994; Dorbath et al. 1996; Thurber et al. 1997) and the North Anatolian Fault in Turkey (e.g., Salah et al. 2007, 2011; Koulakov et al. 2010; Yolsal-Çevikbilen et al. 2012).

Tomography studies in Algeria have been implemented mainly for studying aftershocks at three sites where the greatest number of large earthquakes have been recorded, namely in the region of the 1980 El-Asnam earthquake (Chiarabba et al. 1997), the region of the 1985 Constantine earthquake (Bounif et al. 1998), and the epicentral area of the 2003 Boumerdes earthquake (Ayadi et al. 2008). The seismic tomography of the El-Asnam region (west-central Algeria) shows a complex preexisting structure along which the evolution of a rupture is controlled by the heterogeneity of the crust (Chiarabba et al. 1997). Seismic tomography of the Constantine earthquake source region (eastern Algeria) has yielded the 3D P-wave velocity to depths of 12 km. The resulting seismic model revealed the locations of possible fault planes and a segmentation of the aftershocks into three ruptured segments (Bounif et al. 1998). For the Boumerdes earthquake region (offshore Algeria), the high-resolution study of aftershocks and velocity structure helped to constrain the fault geometry and structure (Ayadi et al. 2008).

## Seismotectonic context

In northern Algeria, seismic activity resulting from the convergence of the African and Eurasian plates is concentrated mainly in the Tellian chain, where moderate to large seismic events have occurred. The most seismically active region is the coastal part of the chain along the junction with the offshore part. Many large events have been recorded in this region, including the most recent earthquake in Boumerdes in 2003, and those documented in historical catalogues, such as the Oran event of 1790, the Algiers events of 1365 and 1716, and the Djidjelli event of 1856.

In the western and central parts of the country, seismicity is related to NE–SW reverse faults or NW–SE conjugate faults, which may be explained by regional compression due to the convergence of Africa with Eurasia. In the eastern part of the country, strike-slip faults are more common, as are found, for example, in the Mila and Guelma basins.

The Beni-Ilmane sequence took place in a transition zone between the Bibans and Hodna massifs, the two main geologic edifices of the Atlasic chain of northern Algeria. More precisely, the event occurred at the western tip of the Jebel Choukchot, one of the southernmost massifs of the Bibans chain.The Choukchot anticline is cross-cut by several E–W- and N–S-trending faults (Figure 1c). Towards the South, the syncline of Beni-Ilmane faces the Jebel Choukchot. It is marked in its NE extension by the existence of a nappes unit called the Selatna tongue (Figure 1b).

As part of the Saharan platform of Algeria, the South Tellian border region, including the Hodna-Bibans region (near Msila) has been of interest to the Algerian Oil Company for several decades. Oil exploration started in this part of Algeria more precisely in the Sidi Aissa region, about 40 km from Beni-Ilmane village, in 1950. Exploration were conducted in two periods: from 1950 to 1962 by Sn Repal (Société Nationale de Recherche et d’Exploitation des Pétroles en Algérie) and from 1963 to the present by Sonatrach (Société Nationale du Transport, de Recherche et de Commercialisation des Hydrocarbures). Seismic profiling and drilling (Figure 1a) performed during these two periods have revealed high hydrocarbon potential in the South Tellian border region. Investigations indicate that oil is contained in the Eocene strata and two reservoir levels have been distinguished. The “A” level is 10–15 m thick and corresponds to the upper Lutetian, and the “B” level (main reservoir) is 40–70 m thick and corresponds to the Thanetian–Ypresian (Kieken 1974 and 1975).

## Data

**Figure 2:**
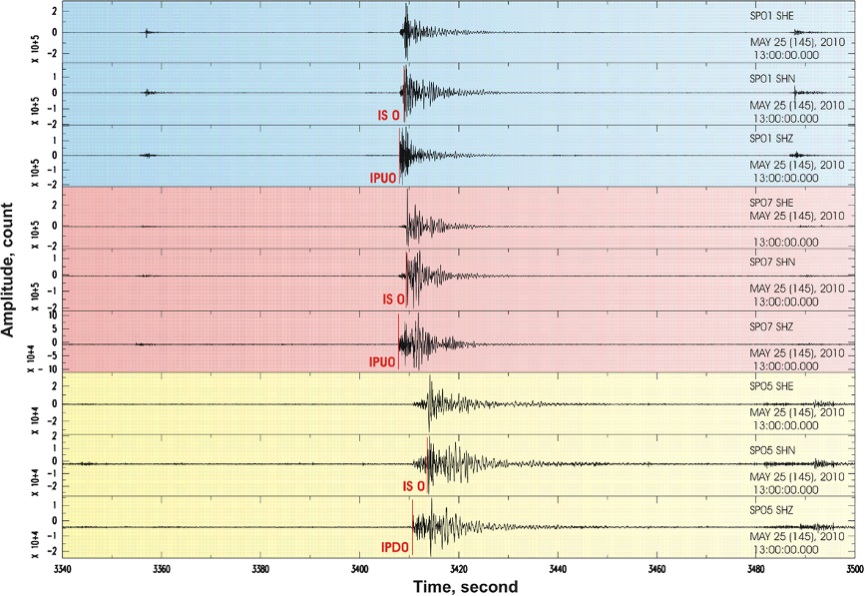
**Example of seismic record of several aftershocks by 3 portable stations with indications of the P and S arrivals is presented.**

The data for the study were recorded by a temporary seismic network consisting of 11 three-component short-period portable stations deployed for about five months in the epicentral area of the 2010 Beni-Ilmane sequence, in addition to the permanent stations of the Algerian seismic network (Figure 1b). Three stations of the permanent network are located at distances of less than 50 km from the epicentral area. For a description of the type and technical characteristics of these stations, see Yelles et al. ( 2013). The arrival times of P- and S waves from local events were manually picked using the Antelope software of Kinemetrics. Figure 2 shows an example of a seismic record of several aftershocks from three portable stations with indications of the P- and S-wave arrivals. To select the data, we used events with no less than 8 picks having the residuals of smaller than 0.8 s. A total of 1406 events were initially selected for the study, corresponding to 12,380 P-wave and 2604 S-wave picks for the interval between 14 and 31 May 2010 (the period of maximum aftershock activity).

## Methodology

We used the Local Tomography Software (LOTOS), described in detail by Koulakov ( 2009) and freely available at http://www.ivan-art.com/science/LOTOS. The version of the code used (version 12) allows for the simultaneous inversion of P-velocities (Vp) and S-velocities (Vs) (and Vp/Vs) and source coordinates.

The input data for the code were the arrival times of local seismicity and station coordinates. The calculations started with the absolute location of sources and estimates of an optimal 1D velocity model. In the version of the code used, the preliminary location of sources is based on a linear approximation of rays, which shortens the calculation procedure. The reference 1D model was defined using a constant Vp/Vs ratio and a fairly simple representation of the P-wave model. We used three to four velocity values at different depths, and velocity was linearly interpolated between the levels. The preliminary location step was performed for many different reference models. The model that produced the minimal root mean square (rms) of residuals and the maximal number of selected data was selected as a reference model for further 3D tomography modeling. In our final selected model, the Vp/Vs ratio is constant at 1.68 and P-wave velocities are defined at four levels (Vp =4.8 km/s at 0 km, 5.2 km/s at 10 km depth, 5.8 km/s at 20 km depth, and 7.0 km/s at 30 km depth).

The tomographic inversion was performed based on an iterative procedure. Each iteration started with source location in the updated 3D model using the bending algorithm for 3D ray tracing. The construction of the parameterization grid was performed only in the first iteration. The nodes of the grid were distributed in the volume of interest according to the ray density. The minimum grid spacing in the vertical and horizontal direction was 2 km. To reduce any effect of grid dependency upon the solution, we performed the inversions for four different grids having different basic azimuthal orientations. Note that the parameterizations used for the velocity representation and 3D ray tracing were completely independent. The inversion was performed simultaneously for P- and S-wave anomalies, source parameters (x, y, z, and t0), and station corrections. The solution was regularized using gradient damping between neighboring nodes. Inversion of the derived matrix was performed using the LSQR algorithm (Paige and Saunders 1982; Nolet 1987). Optimal values of damping and of weighting parameters were defined based on the results of synthetic modeling.

## Modeling and tomography results

The analysis starts with estimating the optimal reference velocity model. We considered a number of different 1D velocity models with quite simple definitions (2–3 velocity values at different depth and constant Vp/Vs ratio) and selected among them a model with provides minimal average residuals and minimum of rejected data. In such model, the Vp/Vs ratio was equal to 1.69 and P-velocities at 0, 10, 20 and 30 km depth were 4.8, 5.2, 5.8 and 7.0 km/s, respectively. Between the depth levels, velocity distribution was linearly interpolated. For this velocity model we selected 1136 events with 9885 P-wave and 2165 S-wave picks, taking into account only those events having more than 8 recorded picks and with the criterion that the residuals computed in the 1D model should not exceed 0.8 s. The values of average residuals and variance reduction during three iteration steps are presented in Table 1. The first iteration corresponds to source location in the 1D starting model. After three iterations, the residuals reduce to 0.089 s and 0.067 s for P- and S-wave data, respectively, which is close to the value of picking accuracy. A significant reduction of residuals (41% and 55% for P- and S data), taking into account the relatively small distances between sources and receivers, indicates the high quality of data and the high variation in seismic properties.

**Table 1:** **Values of standard deviation variance reduction for the P and S data after the step of source locations at different iterations**

Iteration	P-residual deviation, s	P-residual reduction, %	S-residual deviation, s	S-residual reduction, %
**1**	0.153	0	0.150	0
**2**	0.090	40.7	0.070	53.6
**3**	0.089	41.4	0.067	55.4

**Figure 3:**
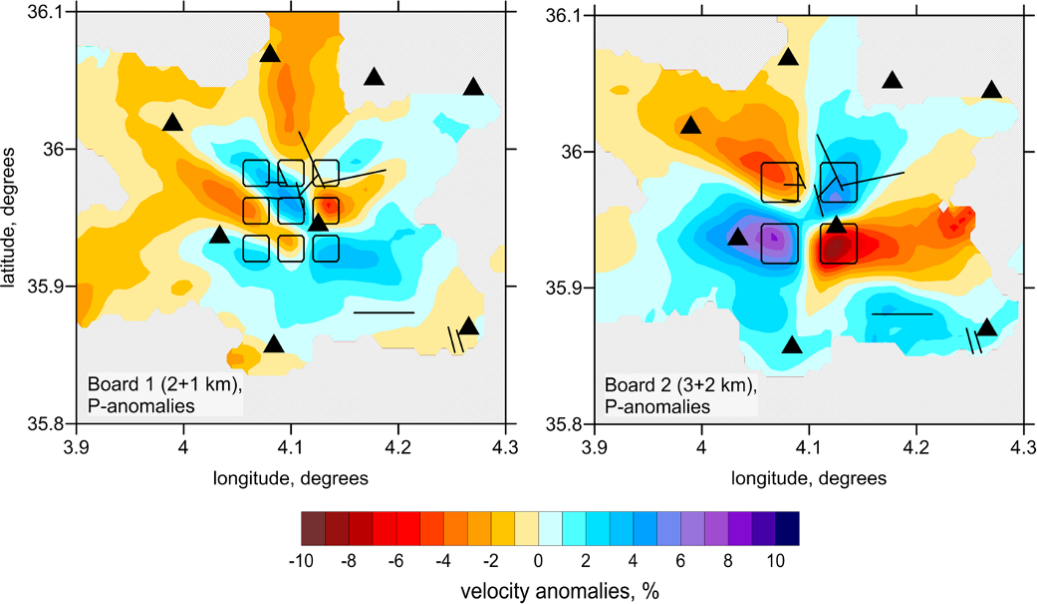
**Inversion results for two checkerboard models composed of 3×3 and 2×2 patterns.** The sizes of anomalies and the spacing is 2 + 1 km and 3 + 2 km, respectively. The reconstructions results are shown for the depth of 4 km. Black triangles indicate seismic stations and the black lines represent the major faults mentioned in the Figure 1b.

**Figure 4:**
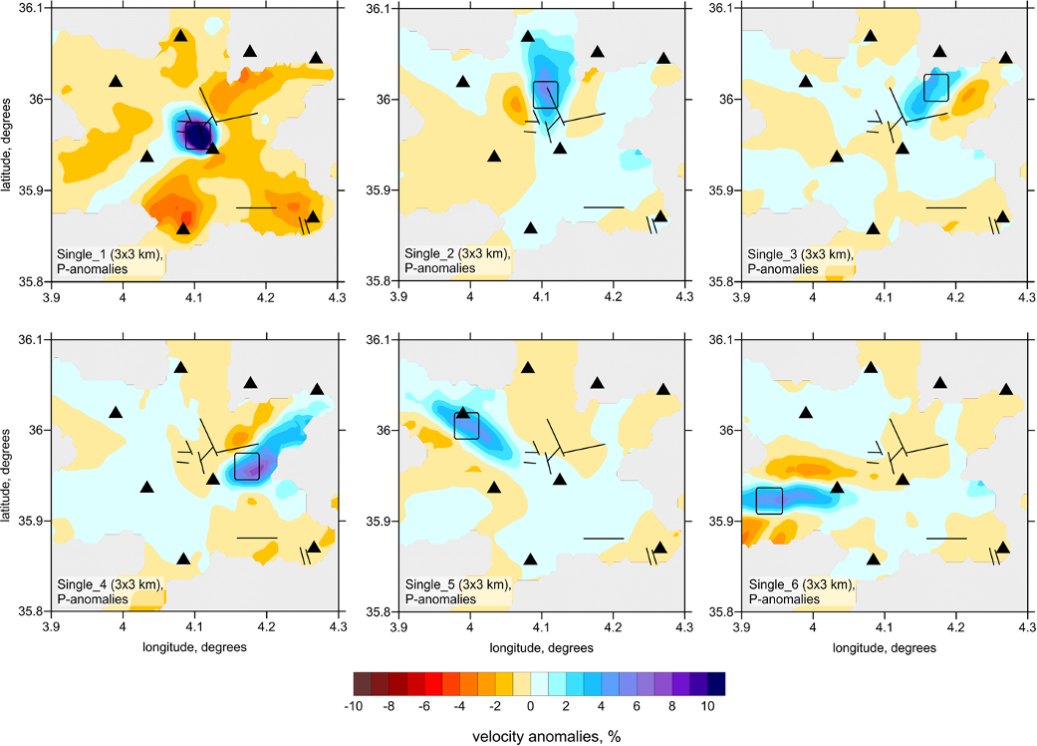
**Examples reconstructions for six models with single synthetic anomalies 3×3 km of lateral size placed in different parts of the study region to further investigate the effect of smearing.** In all these cases, the amplitudes of anomalies were 5%.

**Figure 5:**
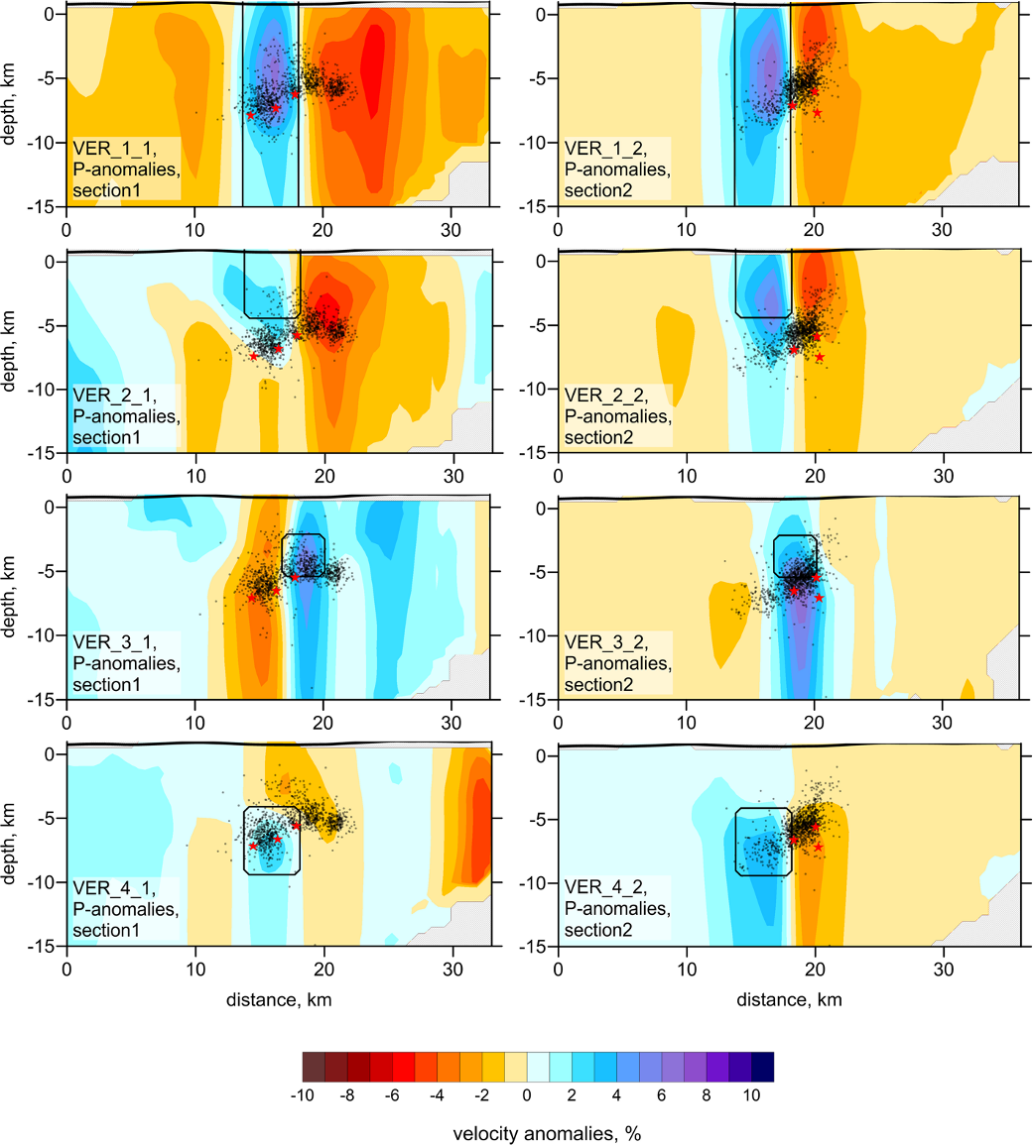
**Examples of reconstructions of four different synthetic models defined along sections 1 and 2 with the position indicated in Figure** 6 **.** Red stars are epicenters of the three main shocks

**Figure 6:**
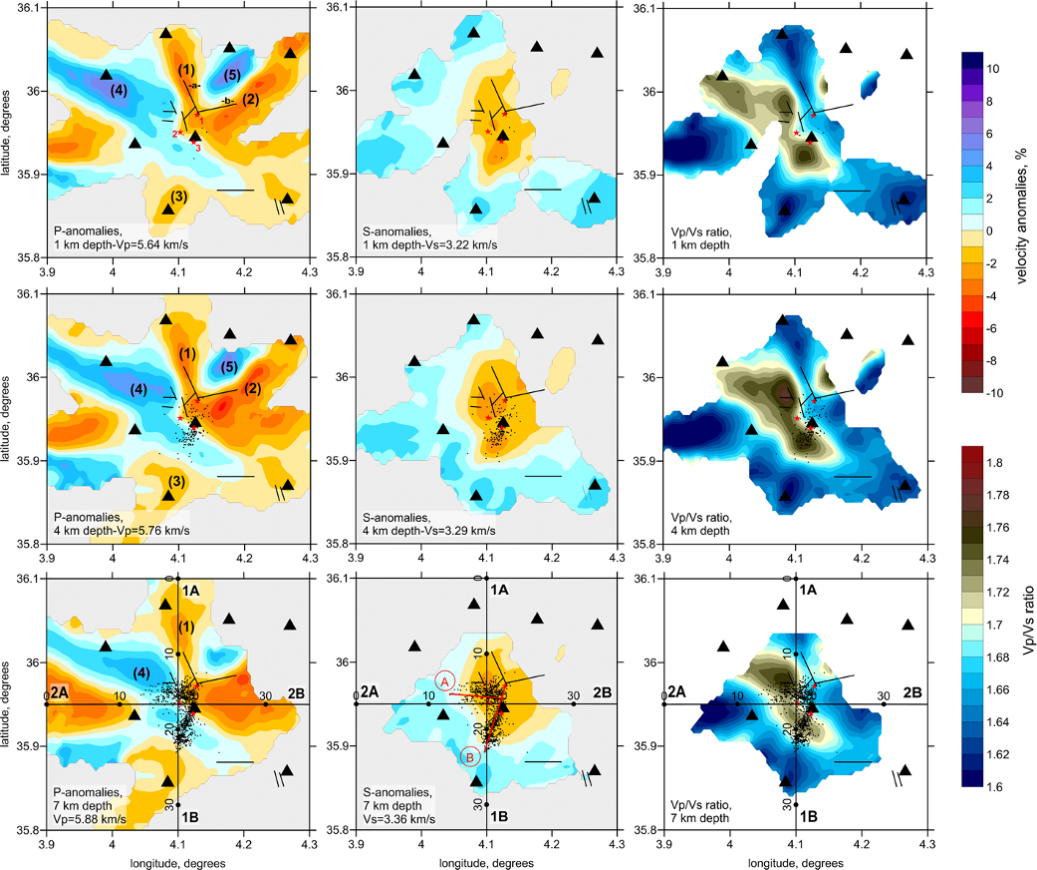
**P, S-velocity anomalies and the Vp/Vs ratio in horizontal sections at 1, 4 and 7 km depth with the corresponding values of absolute reference velocities.** Dots represent the locations of events. Black lines represent the major faults mentioned in the Figure 1b. Black triangles show seismic stations. Red stars are epicenters of the three main shocks. A and B are the two faults proposed according the aftershocks distribution.

**Figure 7:**
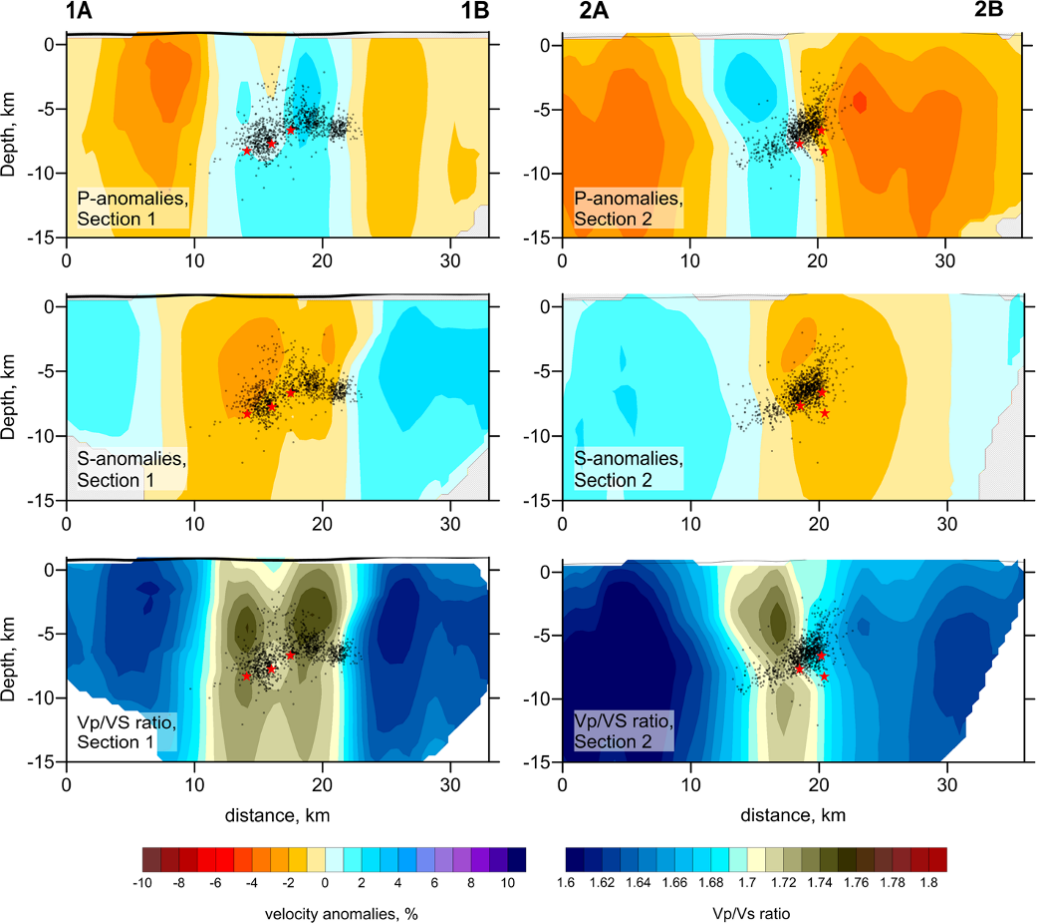
**P, S-velocity anomalies and the Vp/Vs ratio in vertical sections 1 and 2 with the aftershocks relocated.** Red stars are epicenters of the three main shocks.

**Figure 8:**
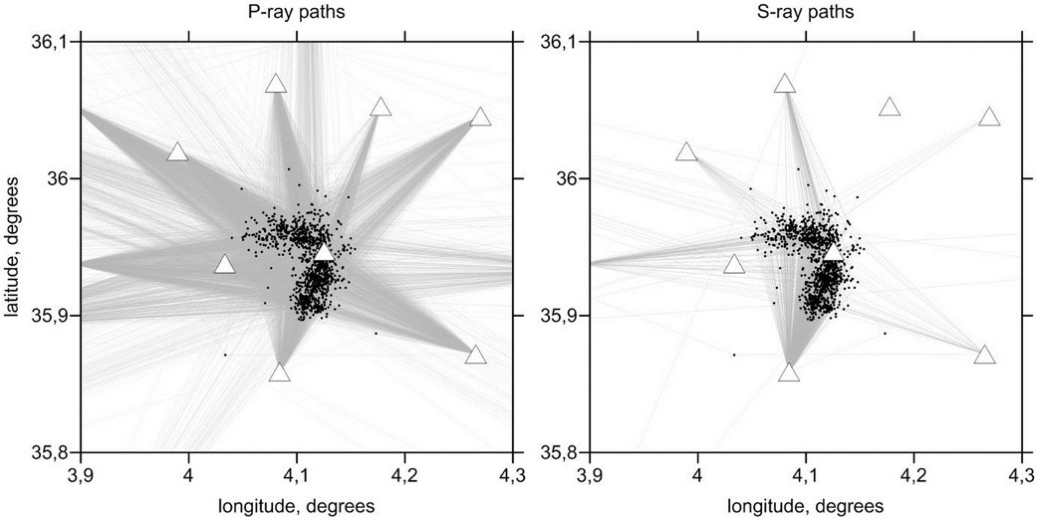
**Path of the P and S rays for the source locations after three iterations of tomographic inversion.** White triangles are the stations; black dots are the events.

To assess the resolution of the results, we performed a series of synthetic tests for which we simulated the conditions of the observed data inversion. In particular, we used identical source–receiver configurations as in the real data experiment. The synthetic travel times were computed using a 3D ray tracing algorithm based on the bending approach. After computing the synthetic data, we ignored the coordinates of the “true” sources, and performed a full calculation workflow, including the source locations, as in the real data case. Thus, in this modeling, we faced the problem of a trade-off between source and velocity parameters, both of which affect the results of passive-source tomography.Figure 3 presents the results of inversion for two checkerboard models composed of 3 × 3 (model “Board 1”) and 2 × 2 (model “Board 2”) patterns. The sizes of anomalies for these two models are 2 km and 3 km, and the spacings are 1 km and 2 km, respectively. The amplitudes of anomalies in all cases were ±5%. In these cases, the anomalies are unlimited with depth. The results of the checkerboard reconstruction show that in the focal area (Figure 3, center of the map), where most earthquakes are located, the boundaries between anomalies in the 2 × 2 case can be clearly discerned. The outer limits of the anomalies are strongly smeared due to the dominant orientations of rays. The test with the 3 × 3 model shows that the 2-km size anomaly can be resolved only in the center of the aftershock area, but the outer anomalies are strongly biased.To further investigate the effect of smearing, we performed 12 different tests with single synthetic anomalies of 3 × 3 km lateral size placed in different parts of the study region. In all these cases, the amplitude of anomalies was 5%, and the anomalies were unlimited with depth. Examples of reconstructions for six of these models are presented in Figure 4. For the case of locating the pattern in the aftershock area (model “Single_1”), the reconstruction reveals the limits of the anomaly correctly. However, for all other cases of placing the patterns outside the central area, the reconstructed anomalies are strongly smeared in the radial directions. This smearing effect should be taken into account when considering the results of data inversion.It is well known than the vertical resolution of passive tomography is usually poorer than the horizontal resolution, which can be explained by the effect of the trade-off between seismic velocity and source parameters. This effect is demonstrated in a series of tests presented in Figure 5. In this case, we defined several synthetic patterns across two vertical sections used for presenting the main results (Figure 6). All these eight plots represent different synthetic models, four for each of two sections; thickness of the anomaly in the direction across the section is 5 km. Here we present the reconstruction results for the P-anomalies. We analyzed different depth intervals of the anomalies, and the purpose of the test was to check whether the existing observation system is able to resolve any differences between these models. In fact, the reconstruction results shown in Figure 5 display a very strong vertical smearing of anomalies, which does not allow the upper and lower limits of the synthetic anomalies to be resolved. These tests show that care is needed when interpreting any vertical changes of structures in real data results.The results of tomographic inversion, which include P- and S-wave velocity anomalies, the Vp/Vs ratio, and the locations of sources, are presented in horizontal and vertical sections in Figures 6 and 7, respectively. We show the anomalies only in areas located at distances of less than 4 km from a nearest node of the parameterization grid which was set according to the ray density. Some anomalies in areas outside the network are related to the ray paths travelling to the remote stations of the Algerian network which were also involved in this study (Figure 8). Note that these outside patterns might be strongly smeared and thus should be considered with prudence. Both the P- and S-wave velocity structures appear to differ in shape from those in our model. The P-wave velocity structures are represented by contrasting patterns with radial orientations. However, as was shown by the synthetic tests, these shapes could be artifacts due to smearing outside the aftershock area. The S-wave velocity model is represented primarily by a single low-velocity anomaly in the central part of the study area that is surrounded by high-velocity anomalies. Interestingly, the amplitudes of the S-wave velocity anomalies are around 5%, which is lower than the value for the P-wave velocity anomalies (7%–9%). The S-wave velocity model shows a much larger reduction in variance and a smaller rms of remnant residuals after inversion than does the P-wave velocity model. Thus, the lower amplitude in the S-wave velocity model cannot be due to poorer quality of the S-wave data and consequent stronger damping in the inversion. On the basis of several inversion trials, we conclude that the obtained amplitudes are realistic. The Vp/Vs ratio model is obtained from the division of the resulting P- and S-wave velocities. This model displays a clear feature of high Vp/Vs ratio values, up to 1.78, in the area of the fault. In the surrounding areas, values are generally low (Vp/Vs =1.62–1.66).The resulting images of horizontal and vertical sections do not show significant variation in structure with depth. However, as was shown by the synthetic tests in Figure 5, the existing data do not allow a robust resolution of the vertical variation.

## Discussion

First of all we should note that the accuracy of source locations is quite high due to the favorable deployment of seismic stations around the aftershock area. We observe a rather well-clustered swarm of seismicity that appears to be clearly aligned in two segments. In map view, the events form a “7”-shaped cluster, with one segment oriented E–W showing a fault segment about 7 km long and 3 km wide (A in Figure 6), and another oriented NNE–SSW showing a fault segment about 8 km long and 3 km wide (B in Figure 6).Two cross-sections were carried out show the vertical distribution of aftershocks and the variation in velocity with depth. Cross-section 1 (1A, 1B in Figure 6) is perpendicular to the first cluster, and cross-section 2 (2A, 2B in Figure 6) is nearly perpendicular to the second cluster. Figure 7 shows the P- and S-wave velocity anomalies and the Vp/Vs ratio in vertical sections 1 and 2 together with the relocated aftershocks. The first cluster (A in Figure 6) is oriented E–W and is distributed on a near-vertical plane 3 km wide (between 13 and 16 km), 8 km long (between 13 and 21 km, see cross-section 2), and between 5 and 9 km deep (see cross-section 1). The second cluster (B in Figure 6) is oriented NNE–SSW and is also distributed on a near-vertical plane 3 km wide (between 18 and 21 km), 8 km long (15 and 23 km, see cross-section 1), and between 3 and 7 km deep (see cross-section 2).

These results are in agreement to those of Yelles et al. ( 2013), who relocated the same data and found that the focal mechanisms of the second cluster (B) show near-vertical left-lateral strike-slip fault planes and that those of the first cluster (A) show a high-angle reverse fault.

The spatiotemporal evolution of the Beni-Ilmane aftershocks as defined by Yelles et al. ( 2013) confirms the three periods of rupture processes that were considered according to the occurrence of the three large main shocks of very similar magnitude (red stars 1, 2, and 3 in Figure 6). The first shock occurred on May 14 on a NNE–SSW-trending, near-vertical, left-lateral strike-slip fault, and may have provoked the second shock that occurred two days later on an E–W reverse fault. The third shock occurred south of the first one nine days after, with the same fault parameters.

No significant differences are observed between the velocity features at different depth levels. This is probably due to poor vertical resolution, as shown by the synthetic tests presented in Figure 5. The structure of P-wave velocity anomalies most likely represents shallow structures and can be associated with the surface geology (black lines in Figure 6). It is widely accepted that P-wave velocity is sensitive mainly to variations in the composition of rocks (e.g., Eberhart‒Phillips et al. 1989; Behn and Keleman 2003). Therefore, the resulting P-wave velocity anomalies may represent geological structures of different origins. The low-velocity anomalies marked (1) and (2) in Figure 6 fit with the fault alignments determined from geological evidence (Baldini 1966). Pattern (1) may coincide with fault “a” and pattern (2) with fault “b” and the syncline of Beni-Ilmane (Figure 1b). These two patterns appear clearly for the first two horizontal sections at depths of 1 and 4 km, but start to disappear from the third section at 7 km depth.No correlation is found between these anomalies and the aftershock distributions that might be explained by a shallow expression of the faults corresponding to the occurrence of seismicity. However, it should be noted that care should be taken with these radial-shaped anomalies, because they can be caused partly by smearing, as was shown by synthetic tests (Figure 4).Anomaly (3) appears to be located at the prolongation of seismicity cluster B and may represent the end of the strike-slip fault where the second phase of aftershock activity occurred. P-wave high-velocity anomalies (4) and (5) may represent rigid blocks of upper crust that are not deformed by regional stresses. These anomalies are located at Jebel Dira and along of the northern part of Choukchot anticline, respectively (Figure 1b).

The S-wave velocity model appears to differ substantially from the P-wave velocity model. In contrast to the compressional velocity, the shear velocity is generally regarded as being related to the fracturing, porosity, and content of fluids (e.g., Domenico 1984; Eberhart-Phillips et al. 1989). We observe an S-wave low-velocity anomaly in the central part of the study region that coincides with the aftershock area (Figure 6). This pattern is especially clear in maps of the Vp/Vs ratio. Relatively high values of the Vp/Vs ratio (~1.78) compared with the surrounding areas (1.62–1.66) probably indicate the presence of high fluid contents. These fluids can be released from deeper levels due to fault movements and quickly migrate upwards. This hypothesis is supported by vertical sections (Figure 7) that show the major Vp/Vs anomalies located above the seismic clusters.

Oil exploration conducted in the South Tellian border region during the 1990s indicated that this area could contain important hydrocarbon reservoirs (Bracene 2002). Two hypotheses for the origin of the oil have been proposed. One considers the bedrock as Turonian (British Petroleum 1995: Petroleum evaluation of the Sour El Ghozlane, unpublished) and the other considers it to be upper Albian (Maxus 1994: Convention Sonatrach-Maxus sur L’Atlas Saharien central, unpublished). Our tomographic investigations through the negative S-wave velocity anomaly shown in Figure 6 seem to reveal oil and gas fields content in these levels.

## Conclusion

The 2010 Beni-Ilmane earthquake sequence represents the most important recent seismic event in the transition seismotectonic zones of the Bibans-Hodna Mountains and the Tellian Chain High Plateaus, where the seismic activity is usually relatively low. This earthquake sequence was marked by three main shocks of very similar magnitude in 10 days, and by strong aftershock activity that lasted for several months. This active seismic phase was triggered by conjugate E–W reverse and N–S dextral faulting.

This study is the first tomographic investigation of the region. We performed inversion using the LOTOS code, which allows for the simultaneous determination of P- and S-wave velocity distributions as well as the relocation of aftershocks. Our selected model was thoroughly verified by various synthetic tests that show an acceptable horizontal resolution in the central part of the study region coinciding with the aftershock area. This is in contrast to the surrounding areas, where the patterns of anomalies are strongly smeared due to the dominant orientations of rays. We also demonstrated the limits of vertical resolution, which can be explained by the effect of the trade-off between seismic velocity and source parameters.

The P-wave velocity anomalies reveal low-velocity patterns that correspond to the fault alignments determined from geological evidence. The derived positive P-wave anomalies may represent rigid blocks of the upper crust that are not deformed by regional stresses.

The S-wave velocity model appears to differ considerably from the P-wave velocity model. We observed a low-velocity anomaly in the central part of the study region that coincides with the aftershock area. This pattern is especially clear in maps of the Vp/Vs ratio. Relatively high values of the Vp/Vs ratio (~1.78) in the central area compared with the surrounding areas (1.62–1.66) probably indicate the presence of high fluid contents. These fluids may be released from deeper levels by fault movements and migrate quickly upwards.

Reports of oil investigations conducted during the 1990s in the South Tellian border region show the existence of hydrocarbon reservoirs (both oil and gas). These reservoirs coincide with the low-velocity anomaly in the central part of the study region tomographic investigations. Then tomographic investigations seem to be a good tool to discuss and reveal presence of oil content in potential hydrocarbon region.

## References

[CR1] Ayadi A, Dorbath C, Ousadou F, Maouche S, Chikh M, Bounif A, Meghraoui M (2008). Zemmouri earthquake rupture zone (*M*w 6.8, Algeria): Aftershocks sequence relocation and 3D velocity model. J Geophys Res.

[CR2] Baldini P (1966). Notice explicative de la carte géologique au 1/50 000 « TARMOUNT » feuille (140).

[CR3] Behn MD, Kelemen PB (2003). Relationship between seismic P-wave velocity and the composition of anhydrous igneous and meta-igneous rocks. Geochem Geophys Geosyst.

[CR4] Beldjoudi H, Guemache MA, Kherroubi A, Semmane F, Yelles-Chaouche AK, Djellit H, Amrani A, Haned A (2006). The Laalam (Bejaia, Northeast Algeria) moderate earthquake (Mw:5.2). Pageoph.

[CR5] Beldjoudi H, Delouis B, Heddar A, Nouar OB, Yelles-Chaouche AK (2011). The Tadjena earthquake (Mw =5.0) of December 16, 2006 in the Cheliff Region (Northern Algeria): waveform modelling, regional stresses, and relation with the Boukadir Fault. Pageoph.

[CR6] Benouar D (1994). The Melouza earthquake of 21 February 1960, Seismicity of Algeria and adjacent region during the twentieth century. Ann. Geofis.

[CR7] Bounif A, Dorbath C (1998). Three dimensional velocity structure and relocated aftershocks for the 1985 Constantine, Algeria (M_s_ =5.9) earthquake. Annali di Geofisica.

[CR8] Bracene R (2002). Géodynamique du Nord de l’Algérie: Impact sur l’exploration Pétrolière. PhD. Thesis. Vol 1.

[CR9] Chiarabba C, Amato A (1994). From tomographic images to fault heterogeneities. Annali di Geofisica.

[CR10] Chiarabba C, Amato A, Meghraoui M (1997). Tomographic images of the El Asnam fault zone, and the evolution of a seismogenic thrust-related fold. J Geophys Res.

[CR11] Domenico S (1984). Rock lithology and porosity determination from shear and compressional wave velocity. Geophysics.

[CR12] Dorbath C, Oppenheimer D, Amelung F, King G (1996). Seismic tomography and deformation modeling of the junction of the San Andreas and Calaveras faults”. J Geophysical Res: Solid Earth (1978–2012).

[CR13] Eberhart‒Phillips D, Han D, Zoback M (1989). Empirical relationships among seismic velocity, effective pressure, porosity, and clay content in sandstone. Geophysics.

[CR14] Kieken M (1974). Thèse ès Sciences, Paris, Pub. Serv. Carte géol. Algérie, nouv. Série, n° 46, t. I, 217 p. et t. II, 281 p. Etude géologique du Hodna, du Titteri et de la partie occidentale des Biban.

[CR15] Kieken M (1975). Thèse ès Sciences, Paris, Pub. Serv. Carte géol. Algérie, nouv. Série, n° 46, t. I, 217 p. et t. II, 281 p. Etude géologique du Hodna, du Titteri et de la partie occidentale des Biban.

[CR16] Koulakov I (2009). LOTOS code for local earthquake tomographic inversion. Benchmarks for testing tomographic algorithms. Bull Seism Soc Am.

[CR17] Koulakov I, Bindi D, Parolai S, Grosser H, Milkereit C (2010). Distribution of seismic velocities and attenuation in the crust beneath the North Anatolian Fault (Turkey) from local earthquake tomography. Bull Seism Soc Am.

[CR18] Nolet G, Nolet G (1987). Seismic wave propagation and seismic tomography. Seismic Tomography.

[CR19] Paige CC, Saunders MA (1982). LSQR: an algorithm for sparse linear equations and sparse least squares, ACM transactions on mathematical software. Trans. Math. Softw.

[CR20] Salah MK, Sahin S, Destici C (2007). Seismic velocity and Poisson’s ratio tomography of the crust beneath southwest Anatolia: an insight into the occurrence of large earthquakes. J Seism.

[CR21] Salah MK, Sakir Sahin S, Aydin U (2011). Seismic velocity and Poisson’s ratio tomography of the crust beneath East Anatolia. J Asian Earth Sci.

[CR22] Thurber C, Roecker S, Ellsworth W, Chen Y, Lutter W, Sessions R (1997). Two-dimensional seismic image of the San Andreas Fault in the Northern Gabilan Range, central California: evidence for fluids in the fault zone”. Geophys Res Lett.

[CR23] Yelles K, Lammali K, Mahsas A, Calais E, Briole P (2004). Coseismic deformation of the May 21st, 2003, Mw =6.8 Boumerdes earthquake, Algeria, from GPS measurements. Geophys Res Lett.

[CR24] Yelles K, Abacha I, Semmane F, Beldjoudi H (2013). The Beni-Ilmane (North-Central Algeria) Earthquake Sequence of May 2010. Pageoph.

[CR25] Yolsal-Çevikbilen S, Biryol CB, Beck S, Zandt G, Taymaz T, Adıyaman HE, AA O (2012). 3-D crustal structure along the North Anatolian Fault Zone in north-central Anatolia revealed by local earthquake tomography. Geophys J Int.

